# Apoptotic effect of novel pyrazolone-based derivative [Cu(PMPP-SAL)(EtOH)] on HeLa cells and its mechanism

**DOI:** 10.1038/s41598-020-75173-8

**Published:** 2020-10-26

**Authors:** Delizhaer Reheman, Jing Zhao, Shan Guan, Guan-Cheng Xu, Yi-Jie Li, Su-Rong Sun

**Affiliations:** 1 Xinjiang Key Laboratory of Biological Resources and Genetic Engineering, College of Life Science and Technology, Xinjiang University, Urumqi, 830046 China; 2 People’s Hospital of Xinjiang Uygur Autonomous Region, Urumqi, 830001 China; 3 Institute of Applied Chemistry, Xinjiang University, Urumqi, 830046 China

**Keywords:** Biochemistry, Cell biology

## Abstract

Pyrazolone complexes have strong anti-tumor and antibacterial properties, but the anti-tumor mechanism of pyrazolone-based copper complexes has not been fully understood. In this study, the possible mechanism and the inhibitory effect of a novel pyrazolone-based derivative compound [Cu(PMPP-SAL)(EtOH)] on human cervical cancer cells (HeLa cells) was investigated. [Cu(PMPP-SAL)(EtOH)] effectively inhibited proliferation of HeLa cells in vitro with an IC_50_ value of 2.082 after treatment for 72 h. Cell cycle analysis showed apoptosis was induced by blocking the cell cycle in the S phase. [Cu(PMPP-SAL)(EtOH)] promoted the loss of mitochondrial membrane potential, release of cytochrome *c*, PARP cleavage, and activation of caspase-3/9 in HeLa cells. Additionally, [Cu(PMPP-SAL)(EtOH)] inhibited the PI3K/AKT pathway and activated the P38/MAPK, and JNK/MAPK pathways. [Cu(PMPP-SAL)(EtOH)] also inhibited the phosphorylation of Iκ-Bα in the NF-κB pathway activated by TNF-α, thus restricting the proliferation of HeLa cells which were activated by TNF-α. In conclusion, [Cu(PMPP-SAL)(EtOH)] inhibited the growth of HeLa cells and induced apoptosis possibly via the caspase-dependent mitochondria-mediated pathway. These results suggest that [Cu(PMPP-SAL)(EtOH)] can be a potential candidate for the treatment of cervical cancer.

## Introduction

Cervical cancer is one of the leading causes of death among women suffering from cancer worldwide, and more than 85% of the deaths due to cervical cancer occur in less developed countries of the world^1^. Since the discovery of anti-tumor property of cisplatin platinum (Pt), commercially synthesized Pt-based drugs have been playing an important role in the treatment of cervical, lung, ovarian, head, and neck cancers^2,3^. Cisplatin, oxaliplatin, and carboplatin have been approved as first-line chemotherapeutics for the treatment of carcinomas in combination with other anticancer drugs^4,5^. However, neurotoxicity, hepatotoxicity, nephrotoxicity, and drug resistance have led to reduced clinical application of Pt-based drugs^6^. Therefore, there is an urgent need for the conceptualization and preparation of new and efficacious anti-cervical cancer drugs with low toxicity.

In recent years, copper complexes have attracted much attention due to their easy synthesizability, high yield and relatively potent biological activity. Since the synthesis of a series of schiff base copper complexes in 1971, it has been reported that schiff base metal complexes have certain effects on cancer treatment^7^. A recent study found that schiff based CdCl_2_(C_14_H_21_N_3_O_2_) complex mediated apoptosis in colon cancer cells by activating the mitochondrial pathway^8^. Polypyridyl copper complex may selectively inhibit tumor cell growth and induce apoptosis in MCF-7 breast cancer cell lines through the mitochondrial apoptotic pathway^9^. Cu(sal)(phen) effectively induced apoptosis in TNBC cells through down-regulation of anti-apoptosis proteins in these cells in vitro and in vivo^10^. A mixed-ligand copper complex [Cu(L)(phen)]·MeOH induced apoptosis in HeLa cells via accumulation of ROS^11^. Recent studies indicated that pyrazolone schiff base compounds might form novel metal complexes with metals such as platinum and copper, and these metal compounds exhibited better anti-tumor and antibacterial activities than pyrazolone^12,13^. In the initial stages of the present study, we synthesized a copper complex of the pyrazolone schiff base, called 1-phenyl-3-methyl-4-propionyl-5-pyrazolone-salicylic hydrazide-copper (II) complex [Cu(PMPP-SAL)(EtOH)]. [Cu(PMPP-SAL)(EtOH)] exhibited significant anti-tumor effects on KB cells in human carcinoma of the mouth floor and multidrug-resistant KBv200 cells, with lower acute toxicity as compared to cisplatin after intraperitoneal injection in mice^14,15^. However, the apoptosis-inducing effect of copper complexes of pyrazolone schiff base on human cervical cancer HeLa cells and its mechanism have not been fully understood.

In the present study, we investigated the growth-inhibiting activity and apoptosis-inducing effect of [Cu(PMPP-SAL)(EtOH)] on cervical cancer HeLa cells in vitro, as well as its effects on the NF-κB signaling pathway induced by TNF-α, and the MAPK cell signaling pathway.

## Results

### [Cu(PMPP-SAL)(EtOH)] exhibits cytotoxic effect on HeLa cell

**Figure 1:**
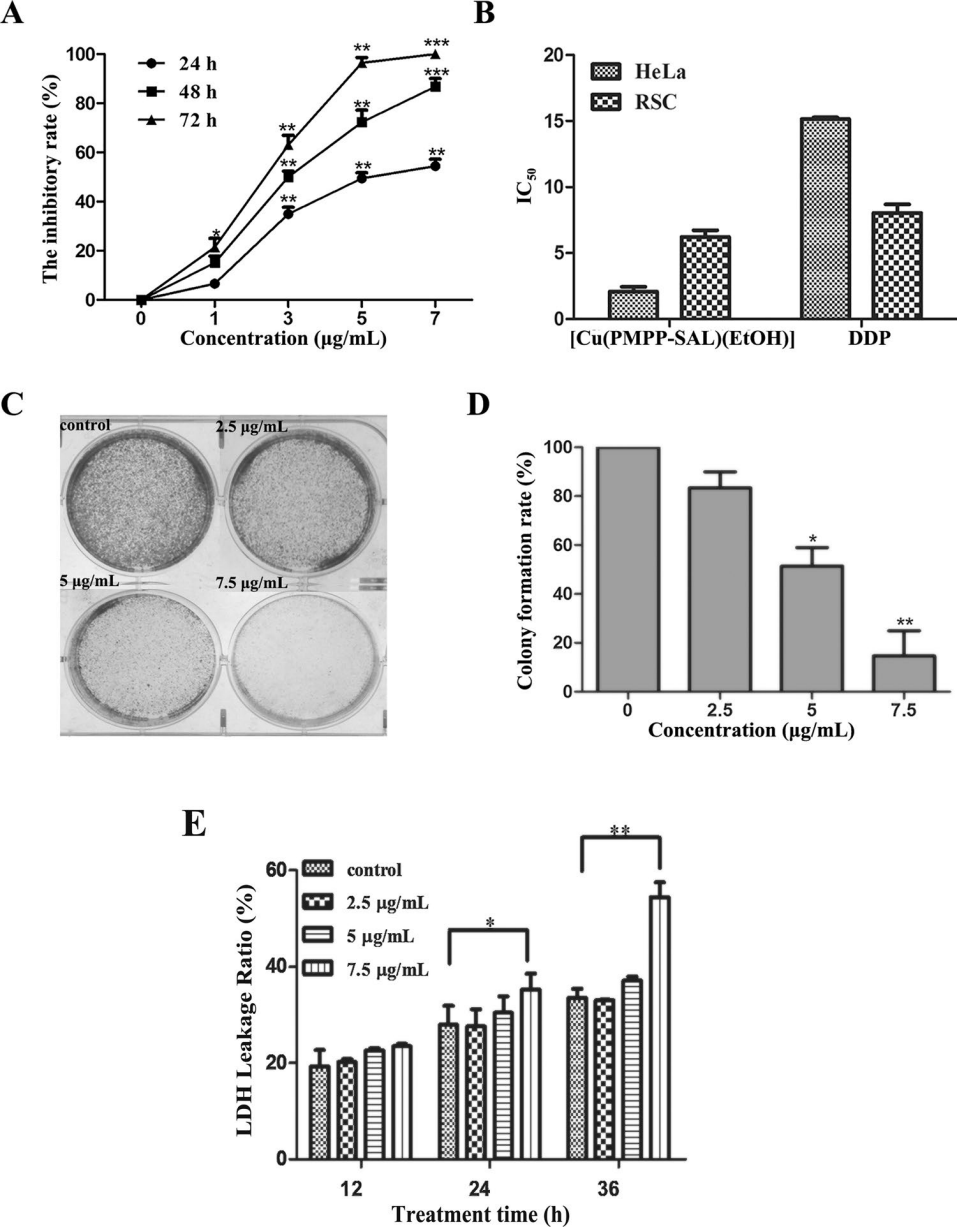
Cytotoxic effects of [Cu(PMPP-SAL)(EtOH)] on HeLa cells. (**A**) HeLa cells were treated with various concentrations of [Cu(PMPP-SAL)(EtOH)] for 24 h, 48 h and 72 h, respectively, then cell viability was measured by MTT assay. (**B**) IC_50_ values of [Cu(PMPP-SAL)(EtOH)] and positive control group DDP in HeLa cells and RSC cells after 72 h treatments. (**C**) The colony formation assay was shown after 24 h treatments. (**D**) Number counts of (**C**). (**E**) LDH leakage level of cells were measured after treating HeLa cells for different time with [Cu (PMPP-SAL) (EtOH)] at the indicated concentration. Data are presented as mean ± SD (n = 3). *P < 0.05, **P < 0.01 vs. control group (DMSO, 0.1% in culture media).

We investigated the effect of [Cu(PMPP-SAL)(EtOH)] on the proliferation of cancer cells by MTT assay. As shown in Fig. 1A,B, [Cu(PMPP-SAL)(EtOH)] caused a time (24, 48, and 72 h) and dosage (0–7 μg/mL) dependent inhibition of proliferation of HeLa cells. The half-maximal inhibitory concentration (IC_50_) of HeLa cells after treatment with [Cu(PMPP-SAL)(EtOH)] for 72 h was 2.082 μg/mL, significantly different from that of HeLa cells treated with positive control cisplatin (DDP) (15.24 μg/mL) (P < 0.05). It was also observed that the IC_50_ value of non-neoplastic RSC cells treated with [Cu(PMPP-SAL)(EtOH)] was 6.218 μg/mL, close to the IC_50_ value (8.048 μg/mL) of non-neoplastic RSC cells treated with DDP. These data suggested that [Cu(PMPP-SAL)(EtOH)] has higher cytotoxicity to cancer cells than to non-neoplastic RSC cells (P < 0.05).

We further preformed a colony formation assay to test the ability of cells to grow under very low density conditions. As shown in Fig. 1C,D, [Cu(PMPP-SAL)(EtOH)] inhibited colony formation of HeLa cells in a concentration-dependent manner. We found that the colony formation rate of cells in 5 μg/mL and 7.5 μg/mL compound treatment group was significantly lower than that in the control group (P < 0.01), thereby confirming that [Cu(PMPP-SAL)(EtOH)] inhibits the colony formation in HeLa cells.

Release of lactate dehydrogenase (LDH) in the culture medium is an enzymatic indicator that signifies loss of membrane integrity, apoptosis, or necrosis of a cell. So an LDH assay was also performed to further validate the cytotoxic effect of [Cu(PMPP-SAL)(EtOH)] on HeLa cells. As shown in Fig. 1E, the leakage rate of LDH from HeLa cells increased in a time and concentration dependent manner after treatment with [Cu(PMPP-SAL)(EtOH)]. Compared with the control group, the release of LDH in HeLa cells was significantly increased after being treated with 7.5 μg/mL [Cu(PMPP-SAL)(EtOH)] for 24 or 48 h (P < 0.05, P < 0.01), suggesting that [Cu(PMPP-SAL)(EtOH)] can cause damage to HeLa cells. In total, these results indicate that [Cu(PMPP-SAL)(EtOH)] had a significant toxic effect on the HeLa cell.

### [Cu(PMPP-SAL)(EtOH)] induced apoptosis in HeLa cells

**Figure 2:**
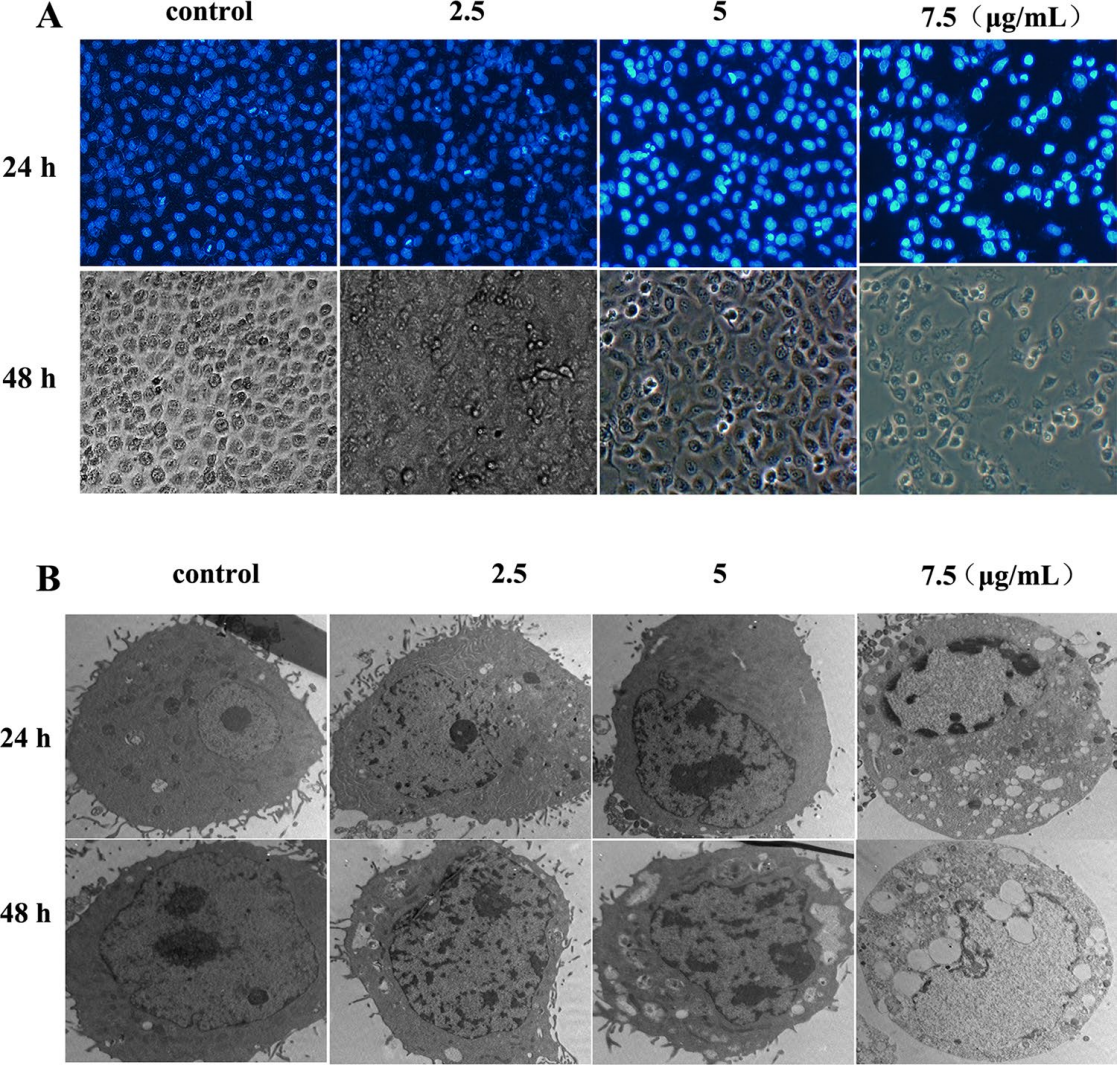
Effects of [Cu(PMPP-SAL)(EtOH)] on cell morphology. (**A**) The HeLa cells were treated with different concentrations of [Cu(PMPP-SAL)(EtOH)] for 24 h, the morphologic changes of cells were observed under a fluorescent microscope after Hoechst 33258 staining. (**B**) The cellular ultrastructure of cells was examined by transmission electron microscope.

We evaluated whether [Cu(PMPP-SAL)(EtOH)] could induce apoptosis in HeLa cells using Hoechst 33258 staining assay. After treatment with [Cu(PMPP-SAL)(EtOH)] (0, 2.5, 5, 7.5 μg/mL) for 24 h, a fewer number of cells and smaller circular morphology of the HeLa cells were observed by microscopy. As shown in Fig. 2A, cells exhibited obvious apoptotic characteristics after treatment with [Cu(PMPP-SAL)(EtOH)] for 24 h, nuclei were condensed and fragmented in the apoptotic cells. Moreover, the ultrastructural alterations were observed under transmission electron microscope. As shown in Fig. 2B, there were no typical morphological changes in control cells. But when cells were exposed to [Cu(PMPP-SAL)(EtOH)] for 24 h, obvious apoptotic morphological changes were observed in these cells. These results suggest that [Cu(PMPP-SAL)(EtOH)] induces apoptosis in HeLa cells.

**Figure 3:**
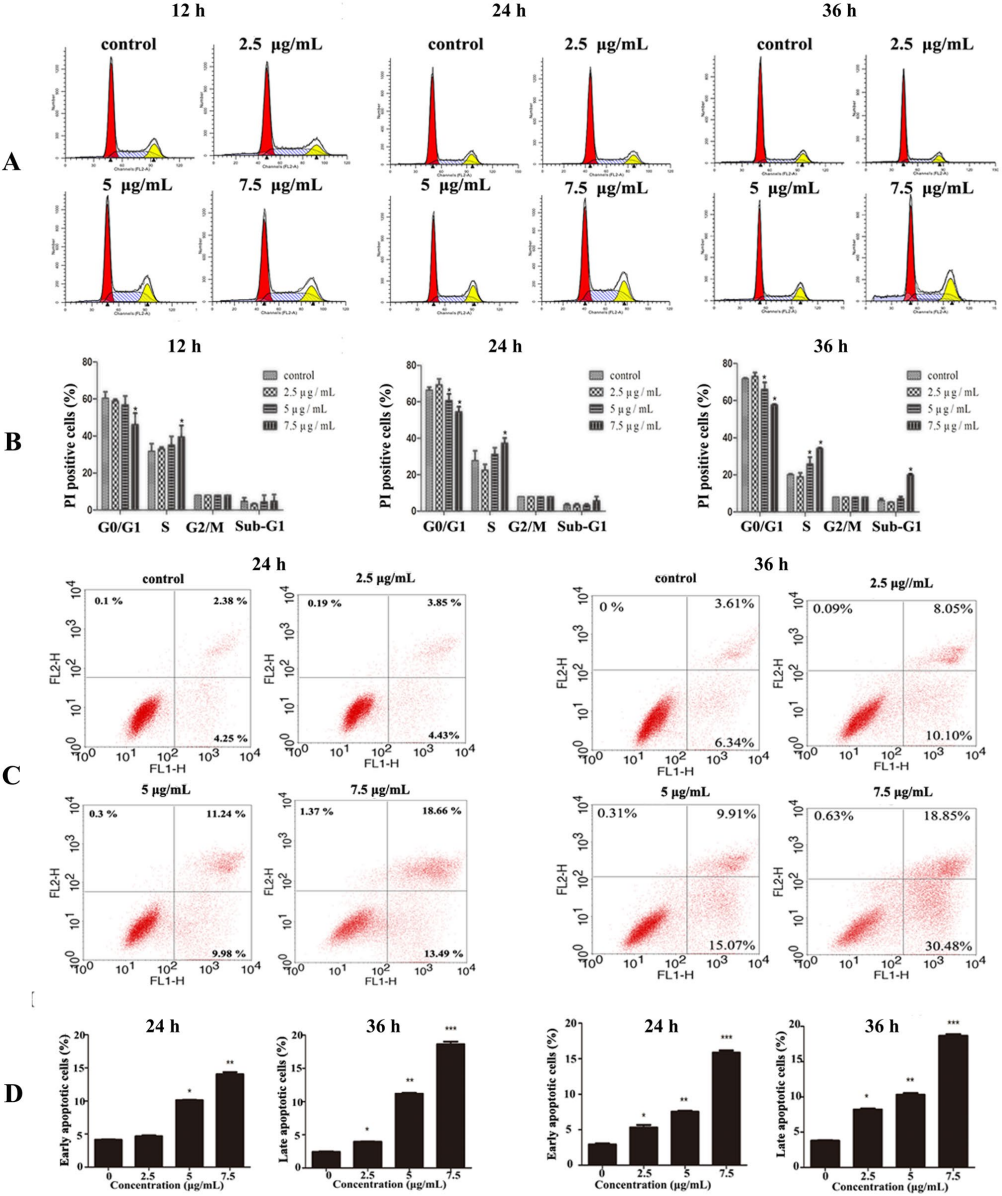
The effects of [Cu(PMPP-SAL)(EtOH)] on cell cycle distribution and apoptosis in HeLa cells. (**A**) The stage of cell cycle of cells were analyzed by using flow cytometry with PI staining after treatment with [Cu(PMPP-SAL)(EtOH)] (0–7.5 μg/mL) for the indicated times. (**B**) Quantitative data of (**A**). (**C**) Apoptosis of HeLa cells were respectively detected with treatment of [Cu(PMPP-SAL)(EtOH)] (0–7.5 μg/mL) for 24 h or 36 h by flow cytometry. Horizontal axis represents Annexin V-FITC intensity and vertical axis shows PI staining. The lines divide each plot into four quadrants: lower left quadrant, living cells; lower right quadrant, early apoptotic cells; upper left quadrant, necrotic cells; upper right quadrant, late apoptotic cells. (**D**) Summary data of early and late apoptotic cells of (**C**) are shown. Data are presented as mean ± SD (n = 3). *P < 0.05, **P < 0.01 *vs*. control group.

After being treated with [Cu(PMPP-SAL)(EtOH)] for 12, 24 and 36 h respectively, the cell cycles of HeLa cells were observed. As shown in Fig. 3A, after treatment for 12 h, the proportion of the cells in the G0/G1 phase reduced from 60.49 ± 3.24% to 46.27 ± 2.42% while that of cells in the S phase was elevated from 31.84 ± 3.98% to 36.25 ± 3.21% as the action dose of the compound increased (Fig. 3B). This indicated that the cells had an S phase arrest that was dose dependent. However, this trend did not change significantly over the increase in action duration, indicating that the action was not significantly time dependent.

Apoptosis was detected via Annexin V/PI double staining. As shown in Fig. 3C, apoptosis was noted in HeLa cells after treatment with increasing concentrations of [Cu(PMPP-SAL)(EtOH)] for 24 h and 36 h. The early apoptotic rate increased from 5.84 ± 0.41% to 31.71 ± 0.87% after treatment with 7.5 μg/mL [Cu(PMPP-SAL)(EtOH)] for 36 h, showing a significant increase over the apoptotic rate after treatment for 24 h (7.5 μg/mL, 14.06 ± 0.43%). The late apoptotic rate also increased from 3.77 ± 0.15 to 18.68 ± 0.32 after treatment with 7.5 μg/mL [Cu(PMPP-SAL)(EtOH)] for 36 h (Fig. 3D). This proves that, [Cu(PMPP-SAL)(EtOH)] increased the apoptotic rate in HeLa cells in a time- and dose-dependent manner.

### Effects of [Cu(PMPP-SAL)(EtOH)] on apoptosis signaling pathway in HeLa cells

**Figure 4:**
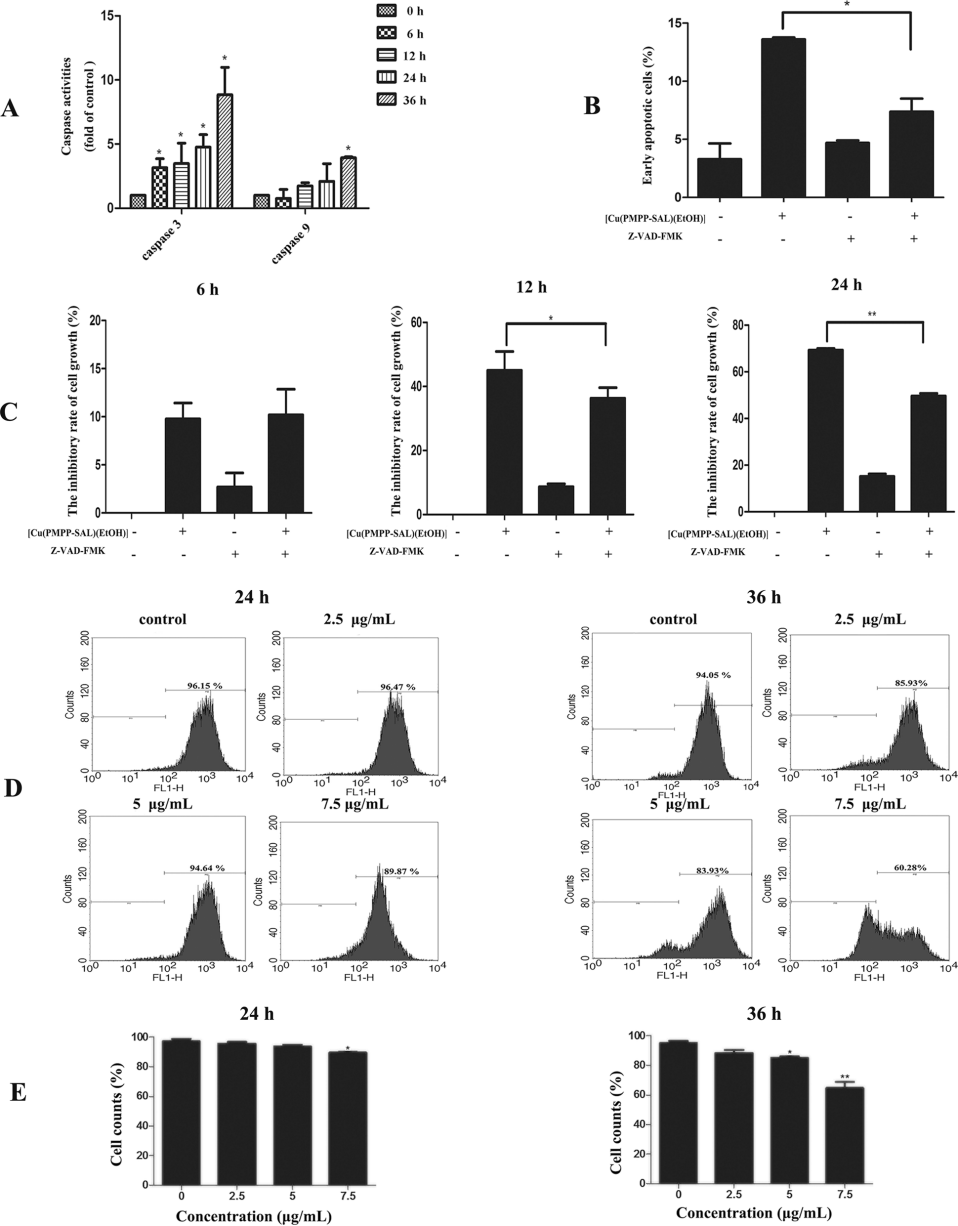
The caspase activity and mitochondrial membrane potential in [Cu(PMPP-SAL)(EtOH)]-treated HeLa cells. (**A**) The activities of caspase-3 and caspase-9 of cells were determined after treatment of [Cu(PMPP-SAL)(EtOH)] (0–7.5 μg/mL) for different times. (**B**) HeLa cells were preincubated with the pancaspase inhibitor Z-VAD-FMK (20 μM) for 1 h before treatment with [Cu(PMPP-SAL)(EtOH)] for 24 h, the early apoptotic rate of cells was analyzed by the Annexin V-PI assay. (**C**) HeLa cells were preincubated with the pancaspase inhibitor Z-VAD-FMK (20 μM) for 30 min before treatment with [Cu(PMPP-SAL)(EtOH)] for 6, 12, 24 h, the growth inhibition of cells was detected by MTT assay. (**D**) After treatment with different concentrations of [Cu(PMPP-SAL)(EtOH)] for 24 h or 36 h, the change of mitochondrial membrane potential of cells were determined with 1 μM rhodamine 123 staining by flow cytometry. (**E**) The corresponding cell counts of (**D**) were calculated. Data are presented as mean ± SD (n = 3). *P < 0.05, **P < 0.01 vs. control.

In order to ascertain whether [Cu(PMPP-SAL)(EtOH)]-induced apoptosis was dependent on caspase activation, we investigated the impact of [Cu(PMPP-SAL)(EtOH)] on the expression of caspase-3 and caspase-9 in treated HeLa cells. Upon treatment of HeLa cells with [Cu(PMPP-SAL)(EtOH)] for 12 h, the expression levels of caspase-3 and caspase-9 were significantly elevated (Fig. 4A). Additionally, pre-treatment of HeLa cells with a caspase pan-inhibitor (Z-VAD-FMK) significantly reduced the growth inhibition capability of [Cu(PMPP-SAL)(EtOH)] in HeLa cells (Fig. 4C). The early apoptotic rate of HeLa cells treated with the compound alone was significantly higher than that of the HeLa cells pretreated with Z-VAD-FMK and subsequently treated with [Cu(PMPP-SAL)(EtOH)] (Fig. 4B), indicating that [Cu(PMPP-SAL)(EtOH)] induced apoptosis of HeLa cells by activating caspase.

We further investigated the changes in mitochondrial membrane potential (Δψm) in treated HeLa cells. As shown in Fig. 4D,E, after treatment with [Cu(PMPP-SAL)(EtOH)] at different concentrations for 24 h and 36 h, the decrease in the Δψm value of cells was significantly higher after treatment for 36 h, as compared with the Δψm values after treatment for 24 h, at concentrations of 2.5, 5, and 7.5 μg/mL for which the values were 88.31 ± 2.18%, 85.02 ± 1.09% and 68.11 ± 3.09%, respectively. Thus, [Cu(PMPP-SAL)(EtOH)] treatment resulted in a decrease in Δψm value of HeLa cells in a dose- and time-dependent manner. Based on these results, it is hypothesized that [Cu(PMPP-SAL)(EtOH)] might induce apoptosis in HeLa cells by reducing the mitochondrial transmembrane potential and further activating the caspase signaling pathway.

**Figure 5:**
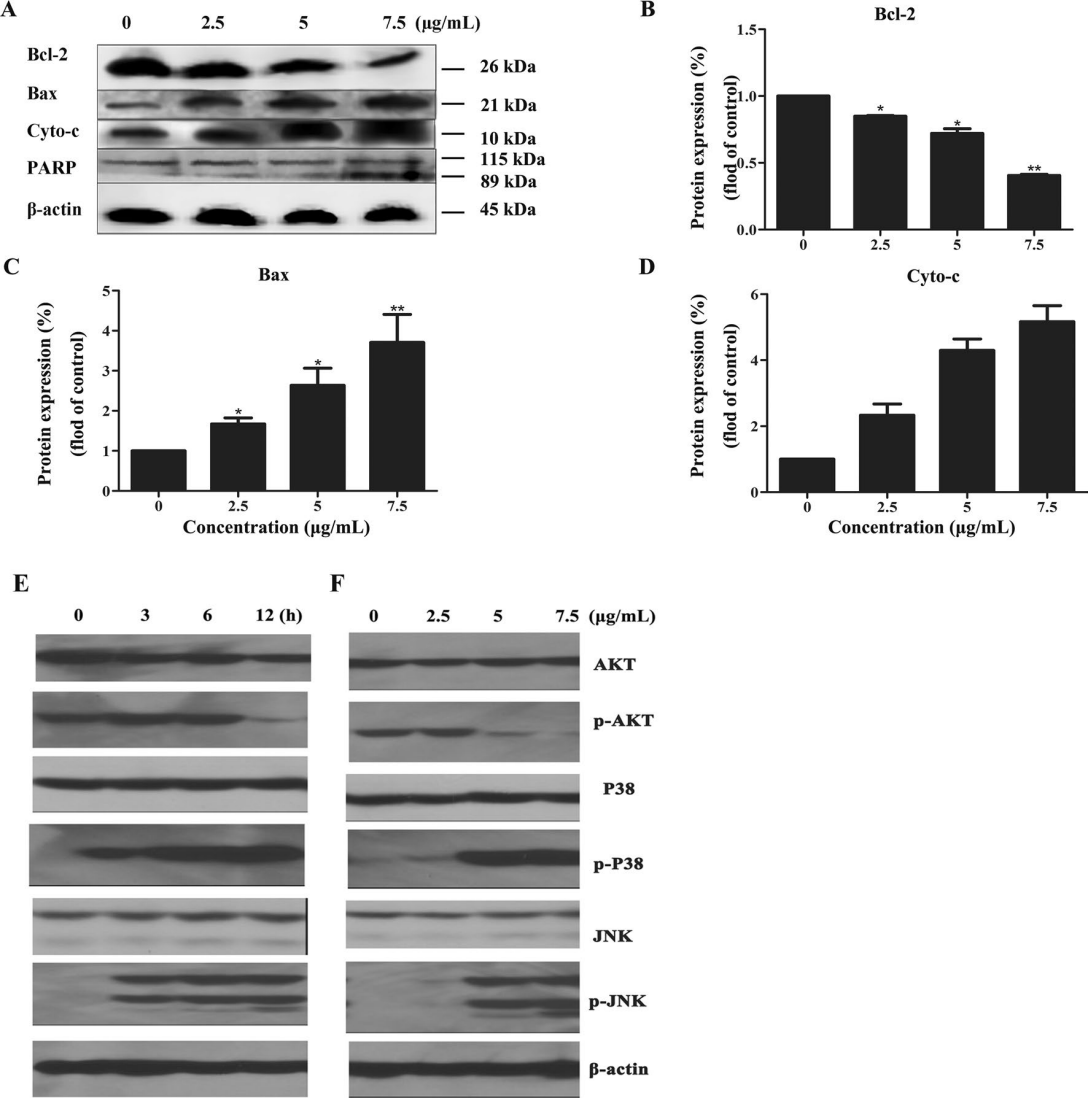
The Effects of [Cu(PMPP-SAL)(EtOH)] on expression of apoptosis-related proteins in HeLa cells. (**A**) After treatment with different concentrations of [Cu(PMPP-SAL)(EtOH)] for 24 h, and the expression of apoptosis related proteins in HeLa cells was detected by western blot. (**B**–**D**) The proteins expression level (fold change relative to control) was analyzed by the ratio of corresponding protein band gray-scale value to internal reference gray-scale value of (**A**). (**E**,**F**) The expression level of p-AKT, p-p38 and p-JNK in HeLa cells was detected after treatment with 7.5 μg/mL of [Cu(PMPP-SAL)(EtOH)] for 0, 3, 6, 12 h (**E**) or with different concentrations of [Cu(PMPP-SAL)(EtOH)] for 24 h (**F**). β-Actin was detected as a loading control for all whole cell extracts. Data are presented as mean ± SD (n = 3). *P < 0.05, **P < 0.01 vs. control.

We further detected the expression level of apoptosis-related proteins using western blot after treatment of HeLa cells with [Cu(PMPP-SAL)(EtOH)]. As shown in Fig. 5, treatment with [Cu(PMPP-SAL)(EtOH)], resulted in cleavage of PARP protein, followed by the release of Cyt *c* protein into the cytoplasm, with a combined effect of significantly reducing the expression of anti-apoptotic protein Bcl-2 and increasing the expression of the pro-apoptotic protein Bax in a concentration-dependent manner (Fig. 5B–D). The aforementioned results indicated that, [Cu(PMPP-SAL)(EtOH)] induces apoptosis in HeLa cells..

In order to measure the inhibitory effects of [Cu(PMPP-SAL)(EtOH)] on growth of HeLa cells, the main signaling molecules in the PI3K/AKT, P38/MAPK and JNK/MAPK signaling pathways were detected via western blot (Fig. 5E,F). The results revealed that, treatment of HeLa cells with 7.5 μg/mL of [Cu(PMPP-SAL)(EtOH)] for 12 h or 24 h resulted in elevated expression of phosphorylated P38 and JNK proteins and reduced level of phosphorylated AKT protein. The results indicate that, the mechanism by which [Cu(PMPP-SAL)(EtOH)] induces apoptosis in HeLa cells may be closely associated with P38/MAPK, and JNK/MAPK signaling pathways.

### [Cu (PMPP-SAL) (EtOH)] inhibited the growth of HeLa cells after TNF-α pretreatment

**Figure 6:**
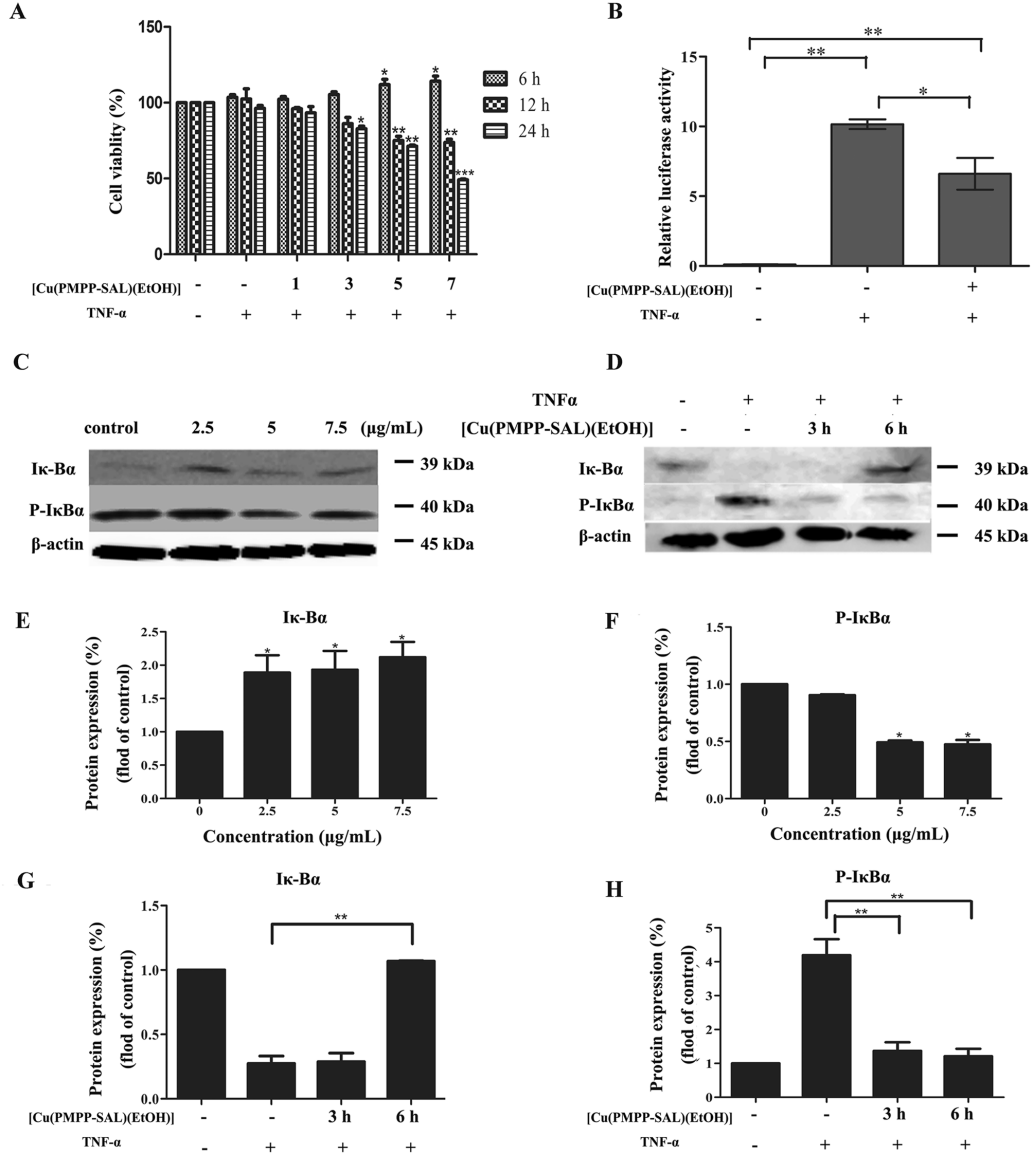
The effects of [Cu(PMPP-SAL)(EtOH)] on expression of NF-κB related proteins induced by TNF-α in HeLa cells. (**A**) After pretreatment of TNF-α, HeLa cells were treated with [Cu(PMPP-SAL)(EtOH)], and the proliferation of cells was examined by MTT assay. (**B**) NF-κB luciferase reporter and control Renilla luciferase reporter vectors were co-transfected into HeLa cells and the relative luciferase activity was measured at 48 h after transfection. (**C**,**D**) The expression of NF-κB-related proteins of cells with or without the TNF-α-pretreatment was detected by western blot after treatment with different concentrations of [Cu(PMPP-SAL)(EtOH)] for 24 h, or with [Cu(PMPP-SAL)(EtOH)] (7.5 μg/mL) for 3 h or 6 h in HeLa cells. (**E**–**H**) The corresponding proteins expression level (fold change relative to control) was analyzed using the ratio of band gray-scale value to internal reference gray-scale value of (**C**,**D**). Data are presented as mean ± SD (n = 3). *P < 0.05, **P < 0.01 vs. control group.

As shown in Fig. 6A, stimulation via TNF-α promoted the growth of HeLa cells, but this growth promoting effect was curtailed by an increase in [Cu(PMPP-SAL)(EtOH)] concentration and duration of treatment. Treatment with 7.5 μg/mL [Cu(PMPP-SAL)(EtOH)] for 12 h significantly inhibited the growth of HeLa cells (P < 0.001), indicating that [Cu(PMPP-SAL)(EtOH)] inhibits proliferation of HeLa cells after TNF-α pretreatment.

In order to verify whether [Cu(PMPP-SAL)(EtOH)] induces apoptosis through the NF-κB signaling pathway, dual luciferase reporter gene system was used to detect the effect of [Cu(PMPP-SAL)(EtOH)] on the NF-κB reporter gene. As shown in Fig. 6B, NF-κB luciferase reporter gene was highly expressed (10.16 ± 0.35) after being stimulated by TNF-α, whereas its expression considerably decreased (6.61 ± 1.13) after treatment with [Cu(PMPP-SAL)(EtOH)], with significant difference between the two groups in terms of data (P < 0.05). The results suggest that [Cu (PMPP-SAL) (EtOH)] inhibits the activation of NF-κB signaling pathway induced by TNF-α.

We further preformed the expression levels assay of Iκ-Bα and P-Iκ-Bα in HeLa cells via western blot after treatment with [Cu(PMPP-SAL)(EtOH)]. As shown in Fig. 6C,E,F, phosphorylation of Iκ-Bα was inhibited as the concentration of [Cu(PMPP-SAL) (EtOH)] increased. Consequently, it can be inferred that [Cu(PMPP-SAL)(EtOH)] inhibits the NF-κB signaling pathway by inhibiting the phosphorylation of Iκ-Bα upstream of the NF-κB signaling pathway. After being pre-treated with TNF-α (10 ng/mL) for 30 min, HeLa cells were treated with [Cu(PMPP-SAL)(EtOH)] (7.5 μg/mL) for 3 h and 6 h, followed by detection of expressions levels of Iκ -Bα and P-Iκ-Bα via western blot. As shown in Fig. 6D,G,H, treatment of HeLa cells with [Cu(PMPP-SAL)(EtOH)] for 3 h and 6 h significantly inhibited the phosphorylation of Iκ-Bα induced by TNF-α, substantiating the theory that [Cu(PMPP-SAL)(EtOH)] inhibits the NF-κB signaling pathway by inhibiting the Iκ-Bα phosphorylation upstream of the NF-κB signaling pathway.

## Discussion

Recent studies have shown the transient metal complexes, such as those containing copper and ruthenium, exhibited anti-tumor effects and pyrazolone can form various complexes with different transient metals to exhibit strong bio-activity^16–20^, but the cytotoxicity of pyrazolone-based copper complexes towards HeLa cells is not clear yet. The anti-tumor mechanism of pyrazolone based copper complexes remains poorly understood. In this study, we found that [Cu(PMPP-SAL)(EtOH)] inhibited the proliferation of HeLa cells and its IC_50_ value was 2.082 μg/mL. The inhibitory effect of [Cu(PMPP-SAL)(EtOH)] on HeLa cells is higher than that of DDP. Although [Cu(PMPP-SAL)(EtOH)] showed inhibitory effect on the proliferation of non-neoplastic RSC cells, its IC_50_ value is much higher than that for cancer cells. The present study showed that, [Cu(PMPP-SAL)(EtOH)] had low toxicity to RSC cells in vitro (Fig. 1B), similar to the results from a study conducted by Kou Chun^15^, which found that the compound had a relatively low acute toxicity in mice. We also found that the inhibition of cancerous cell growth was due to increased apoptosis induced by [Cu(PMPP-SAL)(EtOH)] treatment.

Apoptosis, also known as active death process, is an active and gene-controlled process of cell death for maintaining the stability of the internal environment. At present, there are two main apoptotic signaling pathways, which are the intrinsic mitochondrial pathway and extrinsic death receptor pathway^21,22^. In the mitochondrial pathway of apoptosis, decrease in mitochondrial membrane potential promotes the release of Cytochrome *c* (Cyt *c*) from the mitochondria into the cytoplasm and activated caspase-3, caspase-7, and caspase-9. Therefore, caspase-9 is an important marker of mitochondrial apoptotic pathway^22^. A study found that, a new copper (II) complex with coumarin derivatives promoted caspase-3 activity and induced apoptosis in lung cancer cells^23^. Cu (II) schiff base complexes are believed to promote HeLa cell apoptosis by activating the mitochondrial apoptosis pathway via the activation of caspase-3 and caspase-9^24^. It is possible that upon treatment of HeLa cells with the [Cu(PMPP-SAL)(EtOH)] compound, the activity of caspase-3 and caspase-9 in cells increased threefold. After pretreatment of HeLa cells with caspase pan-inhibitor (Z-VAD-FMK), followed by [Cu(PMPP-SAL)(EtOH)] treatment, significant reduction in the growth inhibition of HeLa cells was observed. After treatment of HeLa cells with [Cu(PMPP-SAL)(EtOH)], the reduced potential of inner mitochondrial membrane may also have caused the breakdown of PARP protein into activated smaller fragments, and may have resulted in lowering the expression of Bcl-2 protein, a Bcl-2 family protein pathway-associated protein, as well as increased expression of Bax protein. The above results suggest that, [Cu(PMPP-SAL)(EtOH)] induces apoptosis in HeLa cells through the caspase-dependent mitochondria-mediated pathway, thereby leading to inhibition of cell growth.

Apoptosis is modulated by complex pathways that involve a series of biochemical regulators and molecular interactions. Early experiments suggested the possibility of interaction between the MAPK pathways and the NF-κB transcriptional cascade^25^. Cell nuclear factor NF-κB is associated with immune responses to cell proliferation, apoptosis, infection, and inflammation^26^. An earlier study had reported that NF-κB in mitochondria might play a regulatory role in cell growth and apoptosis^27^. The activation of NF-κB in tumor cells has been reported to be involved in cell proliferation, blocking of tumor cell apoptosis, promotion of angiogenesis, and enhanced invasion and metastatic capacity of the tumor^28^. In the present study, we found that, [Cu(PMPP-SAL)(EtOH)] exhibited a growth-inhibiting effect on the proliferation of TNF-α-stimulated cervical cancer cells (Fig. 6A). Moreover, we found that [Cu(PMPP-SAL)(EtOH)] inhibited the NF-κB signaling pathway by blocking the Iκ-Bα phosphorylation upstream of the NF-κB signaling pathway (Fig. 6C,D), suggesting that [Cu(PMPP-SAL)(EtOH)] induced cell apoptosis may be related to NF-κB signaling pathway.

MAPK signaling pathway plays an important role in regulating biological mechanisms such as cell proliferation and apoptosis^29^. In the PI3K/AKT signaling pathway, the balance between cell proliferation and apoptosis could be modulated by regulating AKT expression, so as to inhibit tumor cell growth^30^. P38/MAPK signaling pathway has been associated with the induction of apoptosis via various stress signals such as TNF-α, interleukin-1, ultraviolet radiation, hyperosmotic stress, and chemotherapeutics^31^, while the activation of JNK pathway might enhance caspase-3 activity, playing an important role in apoptosis^32^. In the present study, we found that, after treatment of HeLa cells with [Cu(PMPP-SAL)(EtOH)], the PI3k/AKT survival signaling pathway was blocked and the P38/MAPK and JNK/MAPK signaling pathways were activated (Fig. 5E,F), suggesting that [Cu(PMPP-SAL)(EtOH)] may induce cell apoptosis by inhibiting PI3K/AKT and activating P38/MAPK and JNK/MAPK pathways.

In summary, the present study demonstrated that, [Cu(PMPP-SAL)(EtOH)] inhibited the growth of human cervical cancer HeLa cells in vitro and induced apoptosis of HeLa cells through the caspase-dependent mitochondria-mediated pathway. The mechanism of apoptosis may be related to the inhibition of NF-κB and PI3k/AKT pathway, and activation of P38/MAPK and JNK/MAPK signal transduction pathways. These findings showed that, [Cu(PMPP-SAL)(EtOH)] may serve as a potential drug candidate for cervical cancer treatment.

## Materials and methods

### Cell culture

Human cervical cancer (HeLa) cells, non-neoplastic RSC cells, obtained from the China Center for Type Culture Collection (CCTCC, Wuhan, China), were cultured in DMEM medium (HyClone, Thermo) supplemented with 100 U/mL penicillin, 0.1 g/L streptomycin and 10% FBS (TransGen Biotech) at 37 °C under 5% CO_2_.

### Reagents

The reagents and solvents were purchased commercially and used without further purification unless otherwise noted. 3‐(4,5‐Dimethylthiazol‐2‐yl)‐2,5‐diphenyltetrazolium bromide (MTT), dimethyl sulfoxide (DMSO), Hoechst 33258, 2-(6-Amino-3-imino-3H-xanthan-9-yl) benzoic acid methyl ester, RIPA Lysis Buffer and Annexin V-FITC/PI Apoptosis Detection Kit were purchased from Sigma. The Caspase Activity Assay Kit, caspase-3 inhibitor Z-DEVD-FMK, caspase-9 inhibitor Z-LEHD-FMK and caspase inhibitor Z-VAD-FMK, LDH Release Assay Kit were purchased from Beyotime.

### Cytotoxicity assay

The HeLa or RSC cells were seeded respectively in 96-well plates at a density of 5 × 10^4^ cells/mL and 2.5 × 10^4^ cells/mL and cultured for 24 h. After the treatment of various concentrations of [Cu(PMPP-SAL)(EtOH)] for different time, cells were stained with 1 mg/mL MTT and incubated for another 4 h. Then media were removed, and 100 μL DMSO was added to each well to dissolve the purple formazan, and the optic density at 570/655 nm was read.

### Clonogenic assay

HeLa cells were seeded in 6-well plates at a density of 1 × 10^3^ cells/well in the growth medium and cultured for 24 h. Cells were incubated for 24 h with different concentrations (0, 2.5, 5, 7.5 μg/mL) of [Cu(PMPP-SAL)(EtOH)]. Then cells were washed three times with PBS and incubated with DMEM (high glucose) medium for 4 days. After being formed, colonies were washed with PBS and colonies of fewer than 20 cells were discarded. Then each well was added 1 mL of methanol to fix the colonies for 20 min, and 0.1% crystal violet (95% absolute ethanol + 5% PBS) for 15–20 min to stain. Finally, colonies were washed with PBS three times, and the results were scanned with the scanner.

### LDH release assay

The lactate dehydrogenase (LDH) release assay was performed to assess the cytotoxicity potential of the [Cu(PMPP-SAL)(EtOH)]. HeLa cells were seeded in 24-well plates at a concentration of 2 × 10^5^ cells/mL and treated with various concentrations of [Cu(PMPP-SAL)(EtOH)] (0, 2.5, 5, 7.5 μg/mL) for different time (12 h, 24 h, 48 h). The amount of LDH leakage was detected using LDH-Release Assay kit according to the manufacturers’ instruction.

### Morphological assessment

#### Light microscope view

HeLa cells were seeded (2 × 10^5^ cells/well) into a 6‑well plate and cultured for 24 h. After being treated with [Cu(PMPP-SAL)(EtOH)] (0, 2.5, 5, 7.5 μg/mL) for 24 h, cells were incubated in 1 mL of Hoechst 33,258 (0.5 μg/mL) at 37 °C for 5 min. An equivalent volume of DMSO was added to wells that were not treated with [Cu(PMPP-SAL)(EtOH)] as a control. Phase‑contrast images were captured with a fluorescence microscope (Leica Dmirb, Germany) in random microscopic fields at 200× magnification.

#### Transmission electron microscopy

After being treated with [Cu (PMPP-SAL) (EtOH)] at different concentrations of 0, 2.5, 5, 7.5 μg/mL, HeLa cells were fixed with PBS containing 2.5% glutaraldehyde followed by treatment with PBS containing 1% OsO_4_. Then the samples were dehydrated in gradient alcohol, embedded and sectioned. Ultrathin sections were stained with uranyl acetate and lead citrate and then examined with a JEM-1200 transmission electron microscope (JEOL, Japan)^20^.

### Cell cycle analysis

HeLa cells were seeded in 6-well plates at a density of 2 × 10^5^ cells/mL and treated with [Cu(PMPP-SAL)(EtOH)] for 24, 36 h and a DMSO solvent control group was also set. Then cells were collected by trypsinisation, washed with PBS and fixation with 70% ethanol. After being cultured at − 20 °C overnight, cells were washed with PBS and stained with PI/RNase staining buffer for 30 min at room temperature. Finally, the number of cells in the different cell cycle phases was analyzed using a FACSCalibur flow cytometer analysis system (BD FACSCantoTM II).

### Annexin V-FITC/PI assay

2 × 10^5^ cells/mL HeLa cells were seeded in 6-well plates and incubated with [Cu(PMPP-SAL)(EtOH)] ( 2.5, 5, 7.5 μg/mL) for 24 h at 37 °C. At the same time, cells of the same concentration were treated with DMSO solvent as a control group. Both floating and attached cells were harvested, washed twice with PBS and detached by trypsinisation. Then, cells were suspended in 400 μL binding buffer and incubated in 10 μL Annexin V-FITC for 15 min in the dark at room temperature. Next, cells were incubated in 5 μL PI and gently vortexed. Finally apoptosis were analyzed by a FACSCalibur flow cytometer.

### Measurement of caspase activities

The activity of caspase3, caspase9 was detected by (Caspase 3/9 Activity Assay Kit, Beyotime Biotechnology, Shanghai, China) following its instructions. Shortly, the HeLa cells were cultures in 96-well plate and incubated with [Cu(PMPP-SAL)(EtOH)] for 24 h. Then, the cells were treated with suitable caspase9 and 3 reagents and then incubated at 37 °C for 2 h. The optical density for caspase9 and 3 were measured using reader at 405 nm.

To further determine whether the inhibitory effect [Cu(PMPP-SAL)(EtOH)] on HeLa cells growth was affected by caspase pathway, HeLa cells were seeded at a concentration of 1 × 10^5^ cells/mL in a 96-well plate, and 5 μM caspase pan-inhibitor (Z-VAD-FMK) was added to each well. After incubation for 0.5 h, HeLa cells were treated with [Cu(PMPP-SAL)(EtOH)] and continued to be cultivated for 24 h. Then cells were incubated with 20 μL of MTT for 4 h, and the OD at 570/655 nm were detected by microplate reader.

### Determination of mitochondrial transmembrane potential (∆*Ψ*m)

HeLa cells were seeded in 6-well plates and incubated with different concentrations of [Cu(PMPP-SAL)(EtOH)] (2.5, 5, 7.5 μg/mL) for 24 h or 36 h. Then cells were washed with PBS, and incubated with 100 μL of rhodamine 123 (final concentration is 1 μM) for 15 min in the dark. Excess dye was removed by washing with PBS, and measurement of cell-associated fluorescence was performed on FACSCalibur flow cytometer using 488 nm excitation with 505 nm bandpass emission filters.

### Western blot analysis

Apoptotic markers such as Bcl-xl, Bax, Bcl-2, PI3K, P38 and JNK were examined by western blot. Shortly, 1 × 10^6^ HeLa cells were uncovered with [Cu(PMPP-SAL)(EtOH)] or DMSO for 24-h incubation. Then, the proteins were extorted from cells using RIPA buffer. The protein concentration was measured using Pierce™ Detergent Compatible Bradford Assay Kit (USA). Then, the proteins were positioned to 15% SDSP-PAGE and the gel was reassigned to PVDF membranes. The membrane was blocked with 5% bovine serum albumin for 3 h. The membranes were combined with a suitable primary antibody and kept for 24-h incubation at 4 °C. PARP, Cyt *c*, Bcl-2, Bax and β-actin antibodies were from Cell Signaling Technology Co. JNK, phospho-JNK, IκBα, p38, phospho-p38, phospho-AKT and AKT antibodies were obtained from Cell Signaling Technology (Danvers, MA, USA). Then membranes incubated with suitable conjugated horseradish peroxidase secondary antibodies for 1 h. Anti-mouse IgG-HRP and anti-rabbit IgG-HRP were purchased from Santa Cruz Biotechnology Co. Protein bands were detected using the ECL assay kit (Beyotime, Jiangsu, China) and exposed using a Kodak medical X-ray processor (Kodak, USA).

### Analysis of luciferase reporter gene of NF-κB

Luciferase activity was measured using the Dual-Glo^®^ Luciferase Assay kit (Promega). Briefly, targeted cells were transiently cotransfected with specific vectors and phRL (NF-κB-dependent firefly luciferase reporter construct) as an internal control. Cell extracts were prepared, and luciferase activity was measured 48 h after transfection. The NF-κB luciferase activity was normalized to the Renilla luciferase activity.

### Statistical analysis

Data were presented as mean ± standard deviation (SD) of three individual experiments. Student t-tests were performed using the SPSS 17.0, and P < 0.05 was considered statistically significant.

## Data Availability

The datasets generated during and analyzed during the current study are available from the corresponding author on reasonable request.
